# DEFGermplasm: a comprehensive digital platform for forest genomic and phenotype data integration

**DOI:** 10.48130/forres-0025-0009

**Published:** 2025-05-16

**Authors:** Mingjun Du, Meng Liu, Yuhui Li, Huating Hou, Bingyu Chen, Jiarun Gong, Junhong Zhang, Zhengfu Yang, Zaikang Tong, Xiao Han, Huahong Huang, Erpei Lin, Zhengjia Wang, Kean-Jin Lim

**Affiliations:** 1 National Key Laboratory for Development and Utilization of Forest Food Resources, Zhejiang A&F University, Hangzhou 311300, China; 2 Zhejiang International Science and Technology Cooperation Base for Plant Germplasm Resources Conservation and Utilization, Zhejiang A&F University, Hangzhou 311300, China

**Keywords:** Germplasm digitalization, Germplasm conservation, Breeding strategies, Cross-species analysis, Sustainable forestry

## Abstract

Forest germplasm resources are indispensable resources for ecosystem stability and economic value. However, they are increasingly threatened by environmental changes and human activities and urgently need advanced management methods. Concurrently, traditional management methods are unable to cope with the increasingly complex data in the era of advanced information technology. Therefore, we developed DEFGermplasm (https://defgermplasm.com), a digital platform utilizing Flask framework, Python, MySQL, NGINX, Gunicorn, HTML5, JavaScript, JBrowse, SequenceServer, Seaborn, and Echarts, taking the germplasm resources of pecan, hickory, Chinese fir, and phoebe. The platform integrates genomic, transcriptomic, phenotypic, and physiological data, utilizing user-friendly visualization tools to achieve intuitive data presentation. Gene expression heat maps reveal tissue-specific patterns, interactive phenotypic trait visualizations supporting breeding and trait selection, and cross-species analyses uncovering gene conservation and functional adaptation. For example, the WRINKLED1 transcription factor showed roles across nut-bearing and timber species. Additionally, the platform's sequence alignment and homology search tool facilitate comprehensive exploration of gene families, while genome visualization tools provide detailed insights into genomic structures and regulatory elements. By integrating diverse datasets with intuitive visualization tools, DEFGermplasm enhances forest germplasm research, supporting breeding and conservation initiatives. This study underscores the transformative potential of digital platforms in forest genetics and sustainable resource management.

## Introduction

In global forestry, forest tree germplasm resources are foundational, stabilizing ecosystems and providing significant economic value in various fields, such as timber, food, and medicinal materials^[1]^. These resources encompass numerous species, mostly derived from gymnosperms and angiosperms^[2]^, which form the foundation of forest ecosystems and play important roles in maintaining ecological stability and supporting economic activities^[3,4]^. Gymnosperms, such as pine and spruce trees, regulate water and provide wildlife habitats. Their adaptability and resilience make them essential for afforestation and reforestation^[5]^. With their strength and long tracheids, gymnosperms are widely used in the construction and paper industries and in bioenergy production^[6,7]^. Angiosperms (e.g. maple, oak, and fruit trees) offer vast genetic diversity for breeding programs to enhance disease resistance, yield, and adaptability^[8−10]^. They contribute to ecosystem services such as pollination, carbon sequestration, and soil fertility enhancement^[11,12]^. Their hardwood supports the furniture, flooring, and decorative industries because of its density and durability. Angiosperms secondary metabolites benefit the pharmaceutical and biotechnology industries^[13−15]^. Together, gymnosperm and angiosperm germplasm resources preserve genetic information and provide a valuable gene pool for future breeding and improvement efforts, ensuring sustainability in forestry and biodiversity conservation^[5,16]^.

Despite their immense value, forest tree germplasm resources face challenges that threaten their sustainability, including climate change, habitat loss, and overexploitation^[17−20]^. Addressing these challenges requires innovative approaches to ensure preservation and optimal utilization. Advances in information technology and biological big data now deliver the means to modernize conservation efforts to manage resources.

The rapid development of information technology and the advent of the significant data era have dramatically increased the volume of data related to forest tree germplasm resources^[21]^. Traditional resource management and research methods, which rely heavily on field surveys and paper records, are insufficient to handle the complexity and scale of these modern datasets. These methods are inefficient and prone to data loss and damage, limiting their effectiveness in conservation and research efforts^[22,23]^. Modern technologies, such as high-throughput sequencing, remote sensing, and big data analysis, offer transformative possibilities for the digital management of forest germplasm resources^[24,25]^. Digitization enables the systematic, standardized, and visualized management of resource data, enhancing efficiency and accuracy. Advanced visualization tools also allow researchers and managers to analyze and utilize these data intuitively^[26,27]^, enhancing their ability to draw meaningful insights.

Constructing a digital platform for forest germplasm resources will facilitate centralized storage, sharing, and data analysis, providing robust technical support for resource conservation and research. For instance, genomic and transcriptomic data analysis can uncover genetic diversity and evolutionary relationships among species, forming a scientific basis for conservation strategies^[28−30]^. Meanwhile, digitizing phenotypic and physiological data can provide insight into species' adaptability and stress resistance under various environmental conditions, guiding breeding progress and resource improvement efforts^[31,32]^.

Digitization also promotes interdisciplinary integration and accessibility, encouraging collaborative research efforts^[33]^. It enhances data transparency and reproducibility, which are crucial for scientific advancements. Furthermore, digital platforms can employ machine learning and artificial intelligence to predict trends and identify potential threats to germplasm resources^[34,35]^, thereby enabling proactive management and conservation efforts. Utilizing cloud storage and blockchain technology ensures data integrity and security, protecting valuable genetic information from unauthorized access and loss^[36]^.

In recent years, several web-based germplasm resources and modern technologies, particularly high-throughput sequencing, have focused predominantly on crop species such as soybean, maize, oats, rice, and wheat^[37−41]^. However, these platforms have rarely addressed forest tree species, leaving a gap in conserving and utilizing forest germplasm resources. In addition, many of these platforms are difficult to navigate, with information often presented in text form, and they lack robust data visualization tools, making it challenging to interpret complex phenotypic and genetic datasets^[42,43]^. For example, cross-species comparison, which is helpful in understanding the relationship and adaptive traits of species with intuitive visualization, is constrained by existing data management limitations. This challenge highlights the need for a digital platform that integrates various data types, provides advanced data processing and visualization, and supports collaborative research.

To address this gap, we developed the Digital Repository for Characteristic Economic Forest Germplasm Resources Platform (DEFGermplasm; https://defgermplasm.com), a digital platform for forest germplasm resources. DEFGermplasm focuses on edible nut-producing pecan (*Carya illinoinensis* [Wangenh.] K.Koch) and hickory (*Carya cathayensis* Sarg.), and wood-producing Chinese fir (*Cunninghamia lanceolata* [Lamb.] Hook), and phoebe (*Phoebe bournei* [Hemsl.] Yen C.Yang). Pecan, originating from North America, is one of the world's major edible nuts for nutrient-rich kernels and high-quality wood used in furniture manufacturing and construction^[44]^. Hickory, a unique economic forest species native to Zhejiang and Anhui provinces in China, produces protein and fatty acid-rich nuts that are in high demand domestically and exported internationally^[45,46]^. Chinese fir is a fast-growing timber species from southern China that produces high-quality wood for construction, furniture, and pulp^[47,48]^, and also improves soil and water environments^[49]^. Phoebe is a valuable hardwood species from southern China^[50,51]^ with wood known for its dense, fine texture, and aesthetic appeal that is ideal for high-end furniture and architectural decoration^[52]^.

These species span gymnosperms to angiosperms, evergreen and deciduous trees, and timber and fruit trees, ensuring broad applicability across forest germplasm resources. DEFGermplasm integrates genomic and phenotypic data, providing visualization tools and cross-species analytical capabilities to make sense of complex datasets. Built using Python and Flask^[53]^, DEFGermplasm provides a lightweight, efficient platform that integrates diverse data types with minimal resources. It also offers innovative visualization tools and cross-species analytical tools to address challenges in forest germplasm conservation and resource management.

## Materials and methods

### Acquisition of genomics data resources

Genomic and transcriptomic data were collected as core resources of the germplasm platform to support species identification, gene function annotation, breeding improvement, and the study of gene regulatory mechanisms^[54,55]^.

Reference genome sequences, genome structure annotation files in general feature format (GFF), coding sequences (CDSs), protein sequences (PEPs), and transcriptomic datasets were obtained for pecan, hickory, Chinese fir, and phoebe^[56]^. CDSs and PEPs were obtained mainly from previous studies^[57,58]^ or converted from transcriptomic datasets. Transcriptomic data covering various tissues, developmental stages, and treatment conditions of all species were retrieved from published studies and experimental sequencing (Table 1; Supplementary Tables S1–S4). Details of the data processing steps are outlined below. All collected data were available for query and download from the repository.

**Table 1:** Transcriptomics data obtained from species' tissues.

Common name	Scientific name	Tissue	Accession ID
Pecan	*Carya illinoinensis* (Wangenh.) K.Koch	Fruit	PRJNA839178 (NCBI)
Pecan	*Carya illinoinensis* (Wangenh.) K. Koch	Leaf	PRJNA1232222(NCBI)
Pecan	*Carya illinoinensis* (Wangenh.) K. Koch	Root	PRJNA967165 (NCBI)
Pecan	*Carya illinoinensis* (Wangenh.) K. Koch	Flower	PRJNA839160 (NCBI)^[59]^
Hickory	*Carya cathayensis* Sarg.	Fruit	PRJNA687050 (NCBI)^[60]^
Hickory	*Carya cathayensis* Sarg.	Flower stigma	PRJNA810757 (NCBI)^[61]^
Chinese fir	*Cunninghamia lanceolata* (Lamb.) Hook	Leaf	CRA003755 (NGDC)^[62]^
Chinese fir	*Cunninghamia lanceolata* (Lamb.) Hook	Root	
Chinese fir	*Cunninghamia lanceolata* (Lamb.) Hook	Stem	
Chinese fir	*Cunninghamia lanceolata* (Lamb.) Hook	Stem bark	
Chinese fir	*Cunninghamia lanceolata* (Lamb.) Hook	Stem xylem	
Chinese fir	*Cunninghamia lanceolata* (Lamb.) Hook	Male cone	
Chinese fir	*Cunninghamia lanceolata* (Lamb.) Hook	Female cone	
Phoebe	*Phoebe bournei* (Hemsl.) Yen C.Yang	Leaf	PRJNA628065 (NCBI)^[58]^
Phoebe	*Phoebe bournei* (Hemsl.) Yen C.Yang	Root	
Phoebe	*Phoebe bournei* (Hemsl.) Yen C.Yang	Root bark	
Phoebe	*Phoebe bournei* (Hemsl.) Yen C.Yang	Root xylem	
Phoebe	*Phoebe bournei* (Hemsl.) Yen C.Yang	Stem xylem	
Phoebe	*Phoebe bournei* (Hemsl.) Yen C.Yang	Stem bark	

### Phenotypic and physiological data collection

The systematic collection and analysis of phenotypic traits and physiological data provide a scientific basis for the conservation and utilization of forest tree germplasm resources^[63,64]^.

Pecan nuts from 19 varieties (Supplementary Table S5) were collected from Pu'er City, Ning'er County, Yunnan Province (23° N, 101° E), and Lin'an District, Hangzhou, Zhejiang Province (30° N, 119° E) in China^[65]^. Hickory nuts were also collected from Lin'an District (30° N, 119° E, elevation: 119 m)^[60]^. The nuts' length and width were measured to obtain the maximum dimensions in millimeters (mm). At the same time, their weight was determined in grams (g) using an electronic balance. For Chinese fir, the experimental materials were derived from the Chinese fir seed garden (29° N 118° E) in Quzhou City, Kaihua County, Zhejiang Province, and for phoebe, the diameter of breast height was measured in centimeters (cm) and the tree height in meters (m) using diameter tape and laser rangefinders, respectively.

For the edible nut species (pecan, and hickory), the physiological data included moisture content, oil content, soluble protein, soluble sugar, and amino acid content. The drying method was used to measure moisture content^[66]^, and the oil content was measured using the Soxhlet extraction method^[67]^. Crude fat content was determined using acid hydrolysis^[68]^. For the timber species such as the Chinese fir and phoebe, wood properties, including lignin and chlorophyll content, were recorded. Lignin content was determined using the acid hydrolysis method^[69]^. The chlorophyll content was measured using the ethanol extraction method^[70]^.

### Data processing and storage

Data processing and storage are crucial in constructing a digital platform for forest tree germplasm resources. Standardized procedures and advanced techniques were employed to ensure the accuracy, consistency, and usability of the genomic and phenotypic data.

Genomic sequences in FASTA format were obtained from public databases and research groups^[71,72]^. Quality checks were conducted on the raw data to ensure their integrity and accuracy. Preliminary checks and fundamental statistical analyses, such as the total number of sequences, length duplication, and guanine-cytosine (GC) content variation, were performed using the Seqkit toolkit v2.1.0^[73]^. These analyses identified potential issues, including low-quality sequences and abnormal GC content distributions. The redundant sequences were removed using the Seqkit rmdup function to enhance data quality and reduce interference in subsequent analyses. This process improved data reliability by eliminating redundancy and ensuring the independence of genomic data.

Gene ID formats were standardized to ensure consistency across datasets from various sources. Variations in naming conventions, such as suffixes and additional information, were addressed to facilitate downstream analyses. The core components of gene IDs were extracted using the Biopython v1.81 SeqIO module^[74]^. Unnecessary elements, such as version numbers and descriptive information were removed using regular expressions. This standardization ensured compatibility across datasets and minimized errors in gene alignment, functional annotation, and other analyses.

Raw genome annotation files were converted into GFF3^[75]^ format for genomic visualization and annotation tasks. Format compliance was verified using the pyGFF package, which parses and modifies files in GFF format. Feature tags, coordinate information, and attribute columns were checked for accuracy and consistency. Any errors, such as missing key fields or incorrect coordinates, were manually corrected. Redundant or invalid annotation data were removed, and only valid and analysis-relevant information was retained. The standardized annotation files provided a reliable foundation for subsequent genomic visualization and analyses.

Open reading frames (ORFs) of Chinese fir were predicted using TransDecoder software^[76]^, and CDSs were extracted from the ORFs using the getorf tool from the EMBOSS package v6.6.0^[77]^. The CDSs were then converted into PEPs using Transeq^[78]^. This approach streamlined the processing of gene expression data for Chinese fir while ensuring high data quality and accuracy.

For transcriptomic data, raw RNA-Seq data were obtained in SRA format and converted to FASTQ format using the fastq-dump tool of SRA Toolkit v3.0.0. The --split-3 parameter was used to generate paired-end sequencing files, and --gzip was applied to compress the output files for efficient storage. Upon conversion, MD5 checksums were used to verify data integrity. Read quality control (adapter trimming, quality filtering, and length trimming) was performed with fastp v0.23.4^[79]^. Datasets that remained suboptimal were further processed using Trimmomatic v0.39^[80]^. Cleaned reads were aligned to the reference genome using STAR v2.7.8a^[81]^. Before alignment, genome indices were generated via the genomeGenerate mode. STAR performed splice-aware alignment, producing coordinate-sorted BAM files, transcriptome-aligned BAM files, and gene-level read count files. Transcript quantification was conducted using RSEM v1.3.3^[82]^. Reference indices were built with rsem-prepare-reference, and expression levels were estimated using rsem-calculate-expression based on STAR's transcriptome-aligned output. Final expression matrices were generated using rsem-generate-data-matrix. The expression levels from different tissues of the same species were unified and converted to transcript per million (TPM) format to ensure consistency across datasets. To address discrepancies caused by large differences in expression, TPM values were transformed using the formula log_2_(TPM + 1), which is better suited for comparative analysis.

The processing of phenotypic data differed from that of genomic data. Manually collected and processed phenotypic raw data typically contain noise and missing values. Initial checks were performed using Excel to document missing values and identify outliers. Mean imputation^[83]^ was applied for minor missing values, while features with a large number of missing values were excluded. Outliers were identified using the interquartile range method^[84]^ and were either adjusted or removed based on contextual relevance. Data standardization was performed using Python's pandas library^[85]^. Z-score^[86]^ normalization was applied to standardized measurement scales to ensure a mean of 0 and a standard deviation of 1. The Box-Cox^[87]^ transformation was used to normalize the data for indications not following a normal distribution. A correlation analysis was conducted to evaluate the relationships between phenotypic indicators, with significantly impacting target variables retained to reduce redundancy and facilitate visualization.

This hyperspectral study employed 19 pecan varieties sourced from Ning'er, Yunnan Province, and Lin'an, Zhejiang Province, China. Each variety comprised 30 seeds, resulting in a total of 570 samples. Hyperspectral images were acquired using a GaiaField-N17E camera (900–1,700 nm, 5 nm resolution). The samples were uniformly arranged on black cardboard to ensure optimal imaging conditions, and clear images were captured by adjusting the camera parameters^[65]^. MATLAB vR2023b^[88]^ was used to extract the samples' average hyperspectral data, and the corresponding script code is provided in Supplementary Data S1. Various preprocessing techniques, including mean centering, moving average filtering, and multivariate scattering correction, were applied to reduce noise. Subsequently, a machine learning model based on the support vector machine (SVM) model was employed for variety classification. SVM was chosen for its effectiveness in handling high-dimensional spectral data and relatively small sample sizes. Details on the SVM model, kernel function, and parameter optimization have been described in a previously published paper.^[65]^ The SVM model combined with Savitzky-Golay^[89]^ smoothing preprocessing (window size of 21, polynomial order of 3) and principal component analysis^[90]^ for feature extraction (retaining 50 components) achieved the highest accuracy (96.5%) in classifying the 19 pecan varieties. Notably, the classification accuracy reached 100% for four common varieties: Tejas, Jinhua, Shaoxing, and Shoshoni. To use this model, users should upload processed hyperspectral data in .xlsx format (Supplementary Table S6) since raw hyperspectral files are not yet supported. The system accepts a spectral range of 900–1,700 nm, with a 5 MB upload limit for smooth processing. The processed genomic and phenotypic data were stored on the Alibaba Cloud server due to reliable, efficient data storage, recovery, and ensuring long-term data stability access.

### Visualization of genotypic data

Various bioinformatics tools and visualization techniques have been employed to comprehensively analyze forest tree germplasm resources. Essential tools include JBrowse v2.10.3^[91]^, a genome visualization browser, SequenceServer v2.2.0^[92]^, an online sequence alignment tool, and custom-developed tools such as Expression Atlas for gene expression heat maps and Cross Species Expression Atlas for cross-species comparative expression heat maps.

JBrowse*,* a modern genome visualization browser, was integrated into the digital platform to provide a user-friendly interface for browsing and analyzing genome data. Genome sequences (FASTA format) and annotation files (GFF format) for pecan, hickory, and phoebe were prepared for JBrowse by performing data compression and indexing using Samtools v1.13^[93]^ and bgzip v1.13, and the processed genome data were imported into it. The platform enables users to explore genome sequences, gene annotations, and variant sites through a graphical interface and provides an intuitive and interactive tool for genomic analysis.

The Basic Local Alignment Search Tool (BLAST) v2.12.0 was integrated into the platform through SequenceServer to enable sequence-similar searches. The CDSs and PEPs were imported and indexed to ensure fast and accurate alignment. The platforms allow users to upload or input sequences of interest, select alignment parameters and initiate BLAST searches via intuitive user interfaces. The results, displayed in tabular and graphical formats, include alignment scores, similarities, and coverage, with the option of downloading data for further analysis.

Gene expression heat map visualization was developed to enhance the intuitive presentation of data and to improve the user's understanding. This feature visually displays gene expression patterns and biological functions across various tissues. The images correspond to the transcriptome processed earlier. They were hand-drawn and converted into high-quality electronic SVG images using Adobe Illustrator 2023 and served as the background for the gene expression heat maps.

Python v3.10.14 scripts were used to map pre-processed transcriptome gene expression data onto the corresponding tissue images. Gene expression data (transcriptome data) of different tissues were organized and correlated with digital tissue images. The scripts utilized xml.etree.ElementTree to process the resulting SVG files, specifically to read and manipulate the path elements. The maximum expression values were calculated using the math package, defining an appropriate color gradient range for visual representation. The re-module extracted and updated the fill color in the SVG files, calculating new color values based on expression data. The time and os module facilitated filenames and directory management, enabling the deletion of outdated files and the retention of the most recent ones. Heat maps were generated with timestamps to prevent file overwriting.

A query system was developed to enable users to find a gene family name or a specific gene name. This system extracts the corresponding expression data and generates a corresponding visual heat map. For the gene family, expression levels are displayed on tissue images, with color gradients representing expression levels. This system provides users with high-quality heat maps that visually represent tissue-specific gene expression data.

A cross-species gene expression heat map feature was developed to visualize gene expression patterns across multiple species. To better highlight the comparative differences across species, *Arabidopsis* and poplar—two well-established model species with extensive research—were selected for comparison. Gene families were classified using OrthoFinder v2.5.4^[94]^ with BLASTP as the sequence alignment method to identify and categorize gene families across the genomic datasets of pecan, hickory, Chinese fir, phoebe, *Arabidopsis*, and poplar. Additionally, all six species' PEPs were run through the eggnog^[95]^ database to obtain functional annotation information. Transcriptome data for each species were standardized using the TPM method, as described above, to ensure that expression values were comparable across species.

Heat maps were constructed using the Seaborn library in Python^[96]^ alongside hand-drawn tissue images. Each heat map represents the expression level of a gene in a specific tissue, with the color gradient indicating the expression level. Comparative expression patterns of the same gene family across species are displayed through side-by-side or overlay heat maps, providing a collective visual comparison of gene families.

An interactive visualization tool, developed with JavaScript and Python, was implemented to allow dynamic adjustment of displayed gene families and access to detailed expression data. Users can search for a specific gene family and view its expression across different species. The heat maps reveal gene family conservation and divergence by comparing the expression patterns across different species, providing valuable biological information.

### Visualization of phenotypic data

ECharts v5.3.0^[97]^, a dynamic data visualization tool, was used to display phenotypic and physiological data for four tree species. The collected phenotypic data were organized and stored in a custom-built MySQL v8.0.41 database to ensure a structured and queryable format. Python scripts were employed to extract data from the database, followed by cleaning and standardization to ensure consistency and reliability. ECharts was configured to generate various chart types, including line charts, bar charts, and scatter plots, to present phenotypic metrics across different tree species. These interactive visualizations allow users to dynamically select specific metrics and tree species, and adjust the chart views according to their interests. This functionality enables clear and intuitive comparisons of phenotypic traits among the studied species.

To leverage hyperspectral imaging and machine learning advancements in forestry conservation, a hyperspectral prediction model was visualized for pecan varieties. The hyperspectral processing code was consolidated into a single package, which was integrated into the Flask framework for efficient back-end operation. For the front-end visualization, the Swiper component was employed to facilitate interactive operations and enhance the user experience. This integration allows users to interact seamlessly with hyperspectral data and models.

### Website development

The digital platform was developed using a combination of advanced technologies to ensure efficiency and user-friendliness. Python was implemented as the backend language, with Flask v1.1.4 as the framework due to its lightweight and flexible design, which is ideal for constructing complex web applications. For the front end, HTML was used to define the basic structure of the web pages, while CSS and Bootstrap v5.4.0^[98]^ were applied for styling and responsive design. The jQuery v2.1.1 and JavaScript were implemented to enable dynamic functionalities and user interactions, ensuring seamless and engaging user experiences.

The platform was deployed on Alibaba Cloud servers running the Linux operating system (Ubuntu v20.04). Gunicorn v21.2.0, a Python WSGI HTTP server, was utilized to manage dynamic content and handle application requests efficiently, working seamlessly with NGINX to ensure robust performance. NGINX v1.18.0^[99]^, known for its high concurrency handling capability and stability, was configured as the reverse proxy server and static file server to enhance access speed and responsiveness. Systemctl, a Linux system and service manager, was used to start, stop, and restart functionalities, ensuring the stable operation of the website.

## Results

We present the functional capabilities of our platform, DEFGermplasm, demonstrating its utility in genomic research and providing researchers with a robust resource for exploring and interpreting their data.

### DEFGermplasm homepage

**Figure 1:**
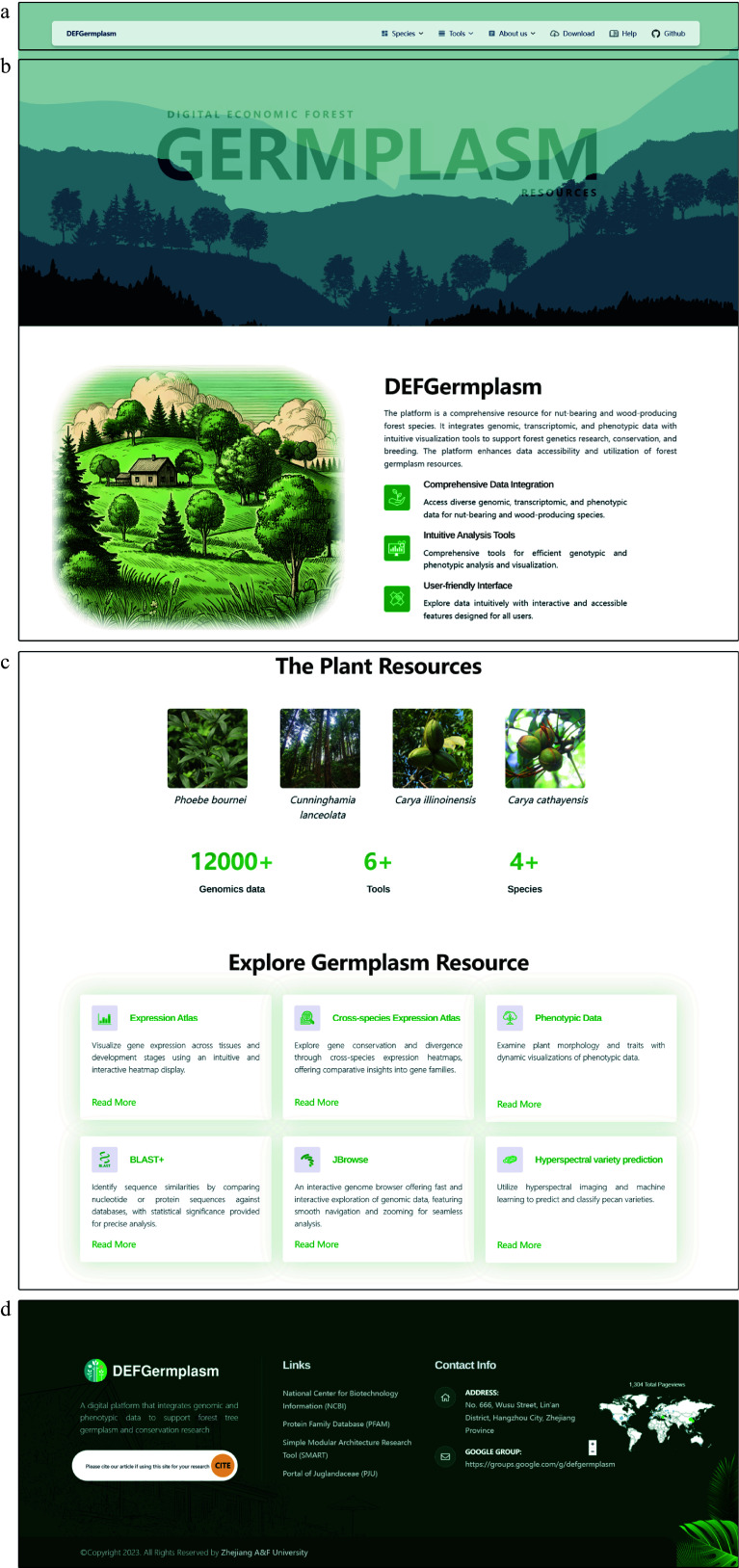
The front page of the Digital Repository for Characteristic Economic Forest Germplasm Resources Platform (DEFGermplasm). (a) The navigation bar offers access to species pages, data downloads, analytical tools, and user help. (b) The homepage features a waterfall effect and silhouettes of species, emphasizing the platform's theme. (c) The main content section displays clickable images of species, offering quick access to their respective pages. This section also summarizes the platform's key datasets and tools. (d) The footer offers links to external analysis sites, website information, contact options, and a plug-in that tracks visitor data.

The homepage of DEFGermplasm (Fig. 1) serves as the central access point, providing users with an overview of the platform's features and tools. It consists of four main sections: the navigation bar, the homepage cover, the main content, and the footer.

The navigation bar (Fig. 1a) offers multi-level menus that allow users to navigate to detailed pages for each featured species. From these pages, users can read, download, and analyze data. The bar also provides access to analytical tools to view genomic information, perform gene sequence alignments, check gene expression levels, and query and download germplasm phenotypic data. Additionally, links to various database platforms previously published by the research team extend the platform's resources. The homepage cover (Fig. 1b) uses a waterfall front-end interaction effect and prominently displays silhouettes of the species, visually emphasizing the platform's theme. This interactive design element enriches the homepage and engages users. The main content (Fig. 1c) concisely introduces the platform. It includes images representing the four species, which users can click on to enter detailed species-specific pages. This section also summarizes the available data and analytical tools, offering users an overview of the platform's core functionalities. The footer (Fig. 1d) contains links to analysis websites, general site information, and contact details. Users can reach out to us via email, and the footer also features a plug-in to track visitor data, enhancing engagement and facilitating feedback.

The design of the DEFGermplasm homepage prioritizes intuitive navigation and a seamless user experience. By integrating key resources and tools into a visually engaging layout, the platform supports the diverse needs of researchers managing and studying characteristic economic forest germplasm resources.

### Genotypic and phenotypic data access and download portal

**Figure 2:**
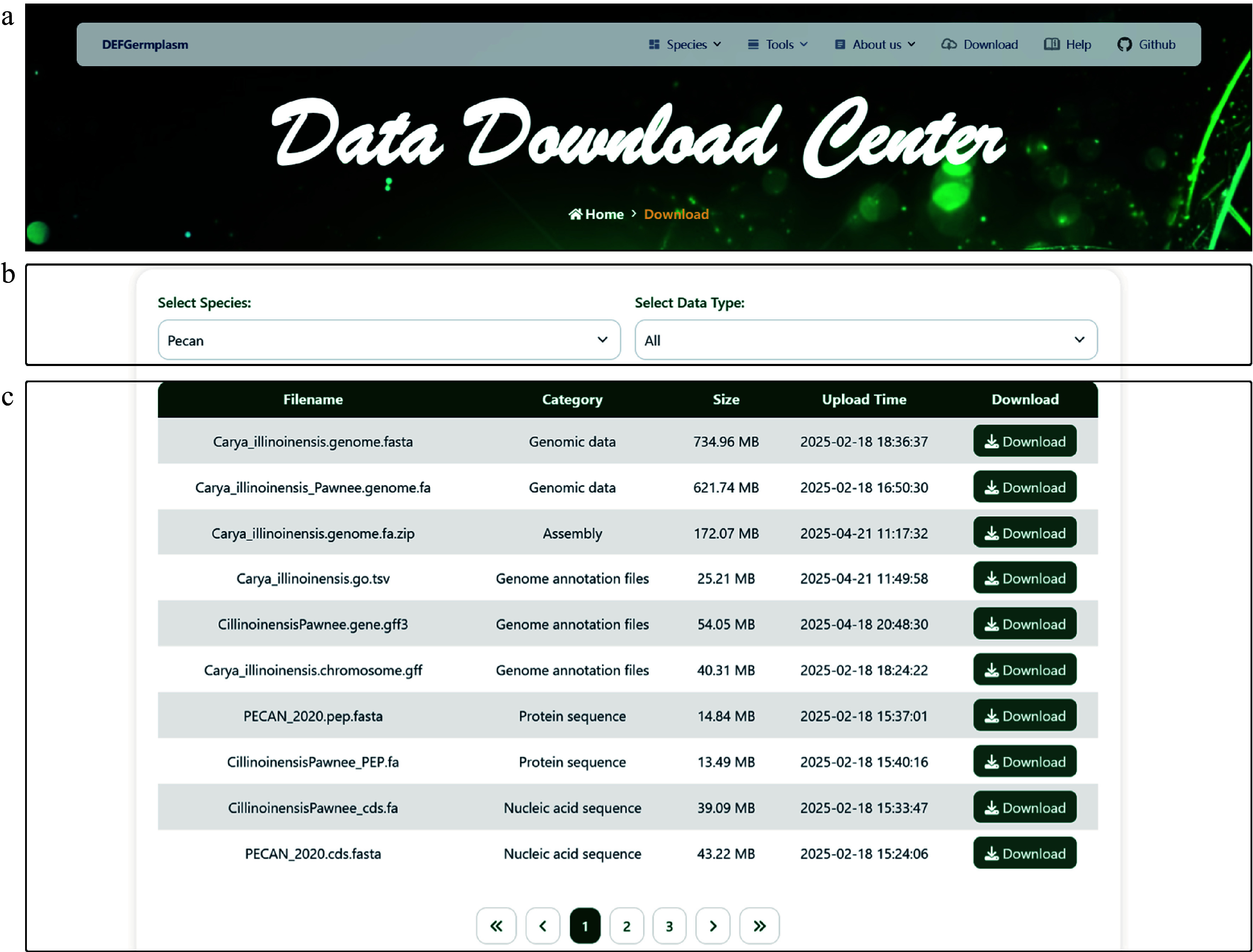
Data Download Center interface. (a) The navigation bar. (b) The data download interface enables users to filter datasets based on specific criteria and download the selected data for further analysis. (c) Presentation of diverse downloadable data for pecan, including genomic, transcriptomic, and phenotypic data.

The DEFGermplasm platform provides access to a diverse range of genotype and phenotype datasets, encompassing genome assemblies, genome annotation, nucleic acid and protein sequences, transcriptome expression, orthologous gene trees, hyperspectral data, and phenotypic trait measurements (e.g., oil content and wood density). All data are available in standardized formats (genomic sequences in FASTA format, phenotypic data in CSV or Excel format) and can be downloaded individually or in bulk via the download interface. Users have the ability to filter datasets by species and data type for large-scale downloads (Fig. 2b). Additionally, DEFGermplasm offers a help section with FAQ resources For troubleshooting or experience sharing, users can connect with the community through the user group (https://groups.google.com/g/defgermplasm).

### Expression atlas: visualising gene expression patterns across tissue

Intuitive visualization is a hub concept of the DEFGermplasm platform. For example, an expression atlas lets users explore and compare gene expression profiles across pecan, hickory, Chinese fir, and phoebe. This feature provides an intuitive way to examine gene expression patterns across various tissues, highlighting tissue-specific expression characteristics and facilitating cross-species comparisons.

**Figure 3:**
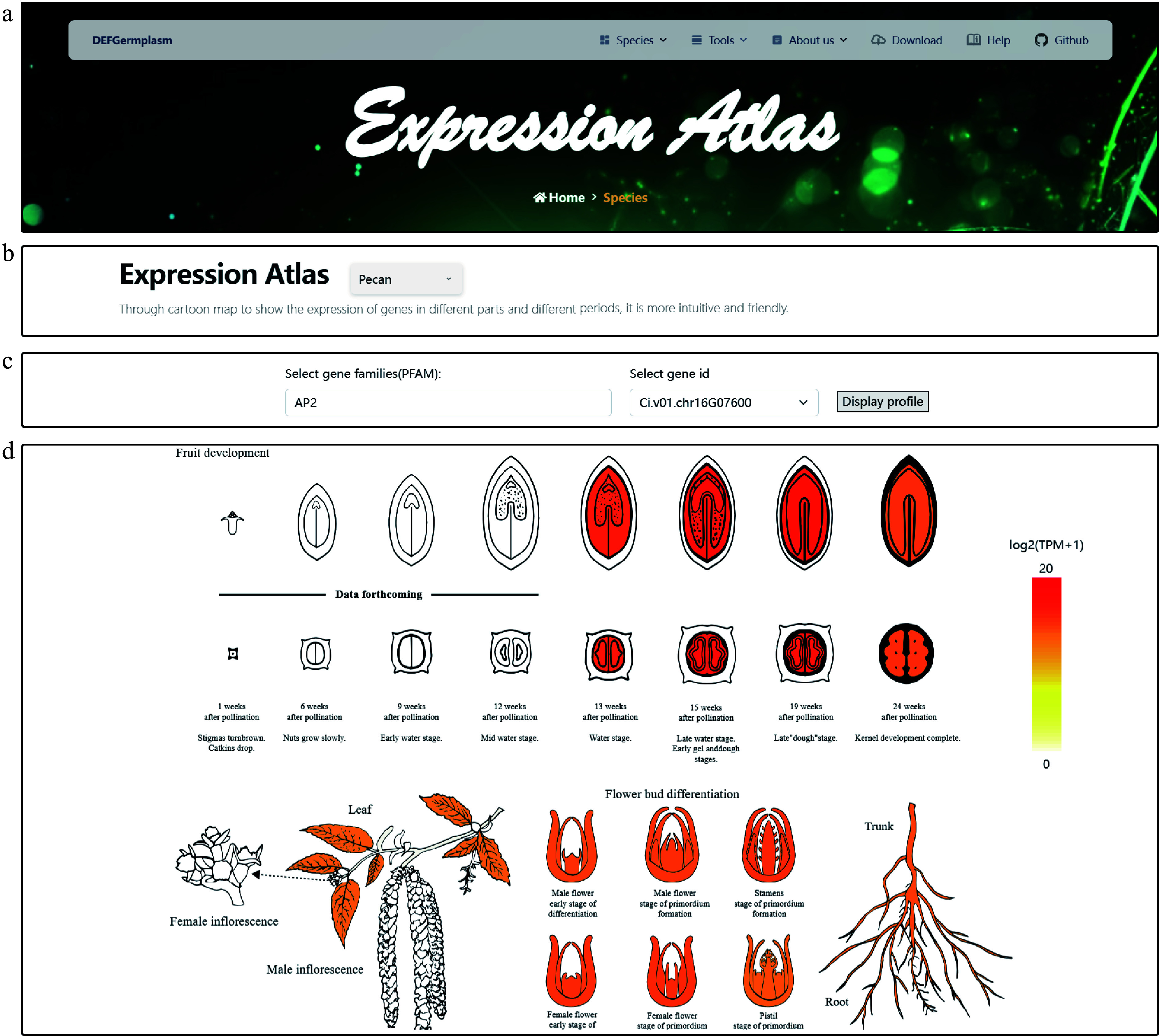
Visualization of the gene expression atlas across different tissues of pecan. (a) Expression atlas logo and navigation bar. (b) Species selection interface for gene family analysis. Users can choose from multiple integrated species, which enables targeted gene family search and comparison. (c) Gene family and gene ID selection interface. Users can select a gene family of interest from a dropdown menu. The platform supports both direct input and fuzzy search to efficiently retrieve specific gene IDs within the selected family. By clicking the 'Display' button, users can generate a tissue-specific gene expression heat map for the selected gene, enabling intuitive visualization of expression patterns. (d) Detailed visualization of gene expression patterns in pecan for gene Ci.v01.chr16G07600, highlighting differences across various tissues. The heat map provides functional insights by illustrating the spatial expression profile of the gene within the plant.

At the platform, users can first select a species of interest (Fig. 3b). After selecting the species, users can then choose a gene family of interest from a dropdown menu, with options for direct input or fuzzy search in the search box. This design enables flexible querying and supports comparative analysis of gene families across different plant species (Fig. 3c). After selecting the gene family, users can choose specific gene IDs to view the corresponding CDSs and PEPs. By clicking the 'Display' button, users can visualize expression levels in different tissues (Fig. 3d). The atlas employs a color gradient with deeper colors indicating higher expression levels. An interactive mouse-based feature allows users to select tissues and view precise expression values. This functionality enables the detailed exploration of tissue-specific expression characteristics and detects sequences with high expression levels. Users can download the results as a PNG file and corresponding expression data in Excel format, providing comprehensive resources for further analyses and functional validation. In addition to the basic search function, we introduced an advanced filtering feature to help users perform initial screening when they do not have a specific target gene. This feature allows them to customize the sorting of transcriptomic data. Users can select the desired expression tissues (e.g., flowers, leaves, roots, and fruits) and specify the number of genes to filter. Upon querying, the genes will be sorted in descending order based on their expression levels. Users can then choose highly expressed genes for heat map visualization or proceed with further analysis.

### Cross-species expression atlas: exploring conservation and divergence

**Figure 4:**
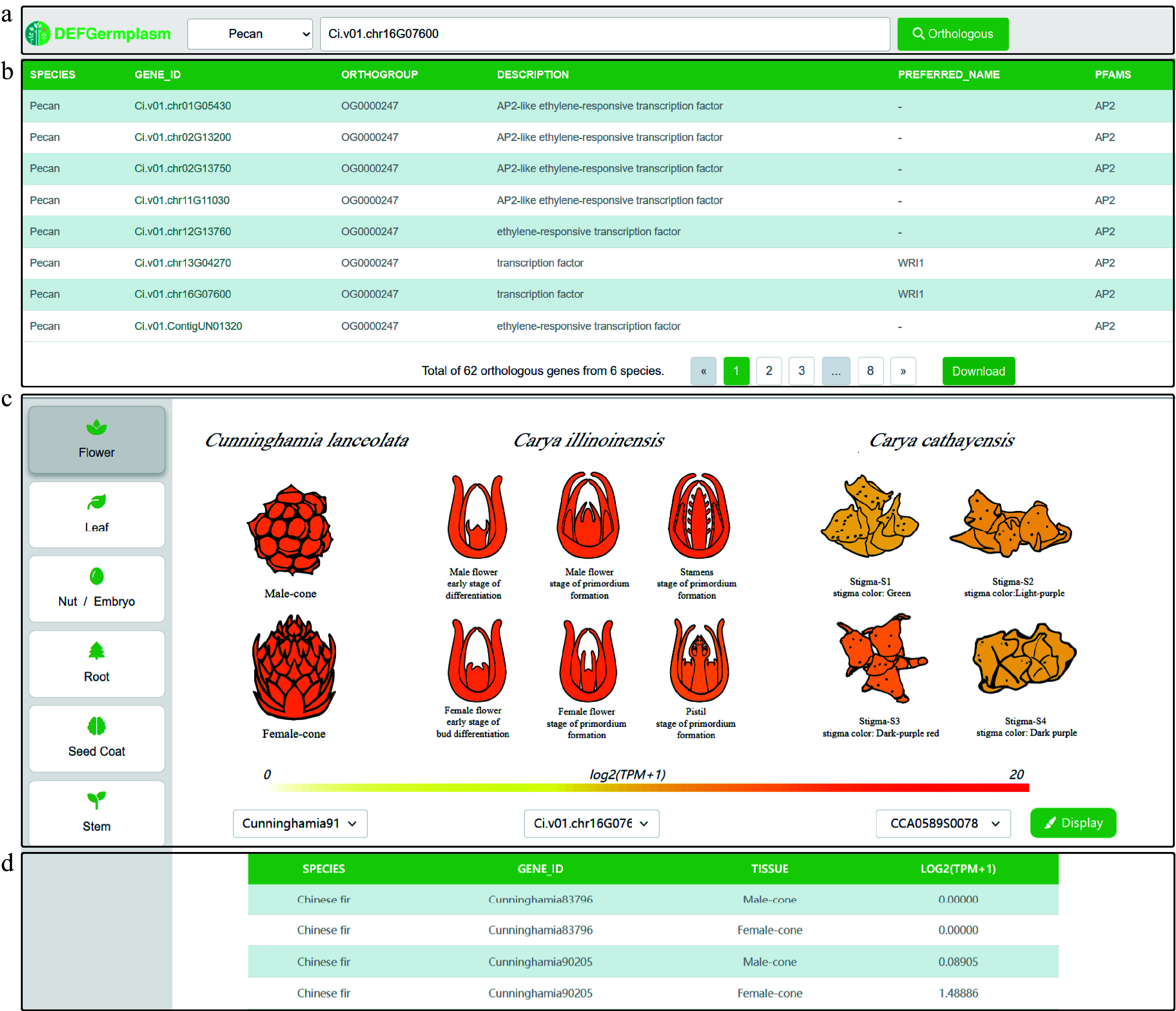
Visualization of cross-species tissue gene expression. (a) Dropdown menu for selecting species and input box for searching genes using gene IDs. (b) Search results table showing orthologous genes, their annotations, and an option to download data for further analysis. (c) Detailed visualization of orthologous gene expression patterns across the same tissue in different species, highlighting tissue-specific differences for functional insights. (d) The summary table shows the gene IDs and their corresponding expression levels in different species, sorted by highest expression level.

Cross-species gene expression heat maps provide valuable insights into the conservation and divergence of gene families by comparing their expression patterns across different species, highlighting both shared and species-specific regulatory mechanisms. Users can select the species of interest from a dropdown menu and input the gene ID in the search box on the right (Fig. 4a). To enhance usability, we implemented a fuzzy search model in the database, allowing users to input partial keywords to narrow down gene queries. By clicking the 'Orthologous Gene' button, users can view the results in the table below (Fig. 4b), which includes a download option for further analysis. Additionally, tissue heat maps for different species are linked to the results, enabling users to select orthologous genes based on species (Fig. 4c). To facilitate easier exploration and filtering, we provide interactive cartoon heat maps that allow users to better understand expression differences, while expression levels are summarized in a table for downstream analysis (Fig. 4d).

### Exploring genomes: interactive visualization with JBrowse

**Figure 5:**
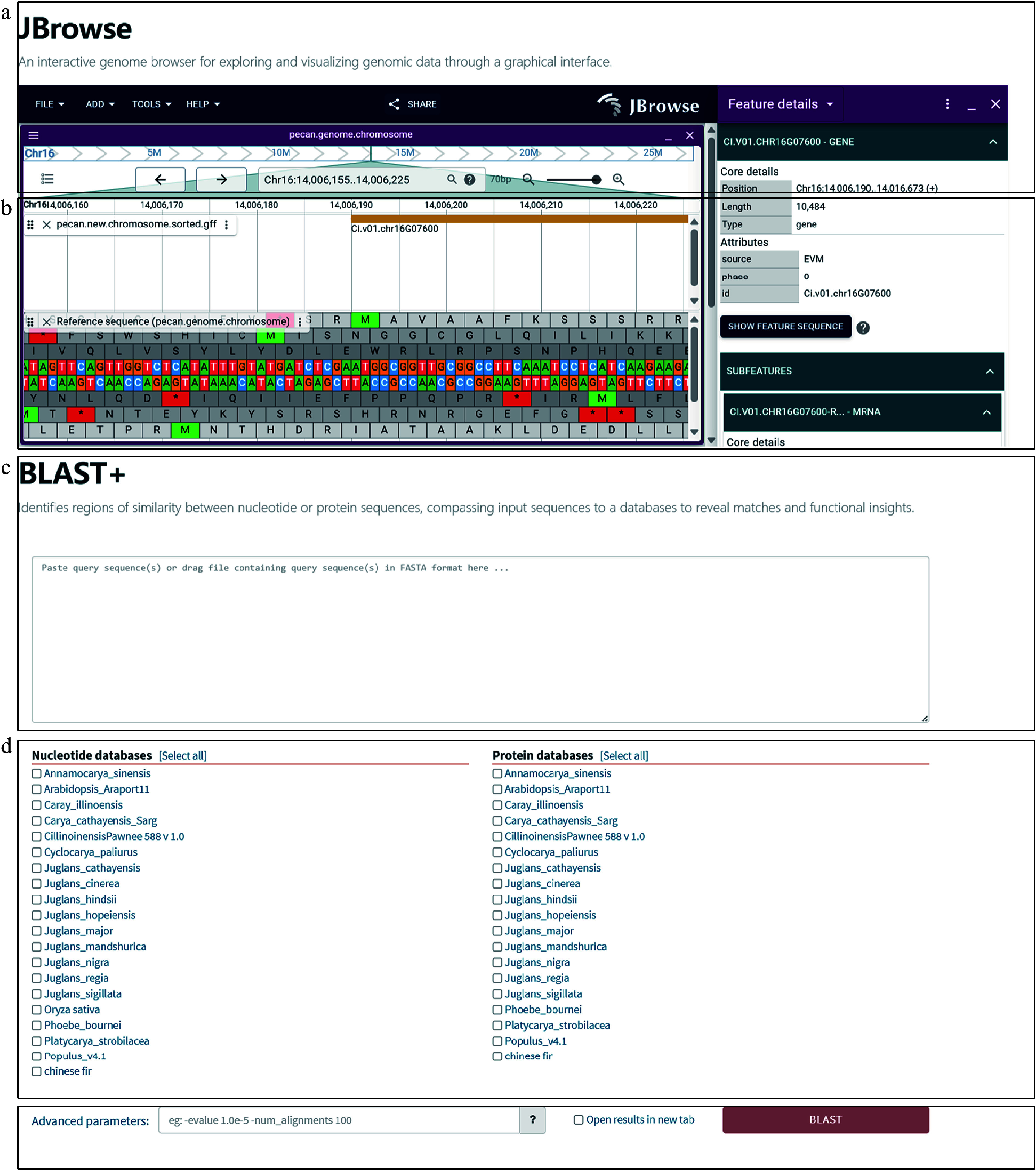
Sequence homology search and interactive visualization of genomic data. (a) Genome browser interface implemented with JBrowse. Users can explore genome structures and annotations through an intuitive graphical interface. (b) Detailed genome view in JBrowse showing gene structures, including exons and introns, along with functional annotations. Users can zoom into specific regions to explore gene features, regulatory elements, and expression data for in-depth genetic analysis. (c) Homology searches are initiated by inputting CDS or PEP sequences through a user-friendly interface that supports both pasting and uploading. Detailed results include alignment scores, match statistics, and sequence similarities. (d) Users can select the desired algorithm (BLASTN or BLASTP) and reference database for comparison. The platform integrates genomic datasets from pecan, hickory, Chinese fir, phoebe, and public databases, enabling multi-species comparisons. (e) Additional parameters, such as e-value thresholds, can be configured to refine search criteria and enhance result precision.

The genome browser, implemented via JBrowse, allows users to explore genome structures and annotations through an intuitive graphical interface. Users can explore gene sequences directly in the genome browser. By entering a gene ID into the input box, they can quickly locate its precise position on the chromosome, enabling intuitive access to gene structure (Fig. 5a). The main view displays detailed genome structures and positional information, allowing users to zoom into specific regions to examine gene sequences, exons, and introns. The functional panel displays comprehensive annotations, including gene names, sequences, exon and intron locations, functional annotations, regulatory elements, and expression data. This detailed view helps users understand specific genomic features and biological roles, facilitating deeper genetic analyses (Fig. 5b). Once users analyze the sequence structure and regulatory elements, they can further investigate gene functions and expression patterns using the platform's integrated analytical tools.

### Sequence alignment: efficient tools for homology search

The sequence homology search tool, integrated via SequenceServer, allows users to upload or input gene sequences efficiently for similarity searches. The user-friendly interface provides detailed results, including alignment scores, match statistics, and sequence similarities (Fig. 5c). The tool supports CDSs and PEPs analysis, offering flexibility for diverse research needs.

Users can initiate a query by inputting CDSs or PEPs (Fig. 5c), selecting an algorithm (BLASTN or BLASTP), and choosing a database for comparison (Fig. 5d). Our platform integrates genomic data from pecan, hickory, Chinese fir, phoebe, and published datasets, simultaneously allowing multiple comparative analyses. Additional options, such as e-value thresholds, refine search parameters for greater precision (Fig. 5e).

The results interface provides a detailed overview of alignment outcomes, featuring graphical visualizations, such as a chord diagram of sequence similarities, an alignment hit summary, and a length distribution of matching sequences (Supplementary Fig. S1). High-confidence matches and alignment data can be downloaded in tabular and XML formats, along with all analysis charts. These results enable researchers to map query sequences to species in the database and explore their features through genome visualization tools.

### Exploring phenotypic traits: interactive visualization tools

The phenotypic data visualization module (Supplementary Fig. S2) provides an intuitive and interactive platform for exploring phenotypic traits across featured species. Users can browse 19 pecan varieties and select specific varieties by clicking on their images (Supplementary Fig. S2a). Upon selection, users are redirected to a detailed phenotypic data display page. Bar charts intuitively show metrics such as nut length, width, weight, moisture content, oil content, soluble sugars, and soluble proteins (Supplementary Fig. S2b). Users can select three pecan varieties to compare morphological differences through images and explore their content differences in more detail through visualization icons (Supplementary Fig. S2c). This provides a more convenient and efficient approach to utilizing germplasm resources. For Chinese fir and phoebe, graphical representations display data for tree height, trunk diameter, crown width, lignin content, and chlorophyll content (Supplementary Fig. S2d, Supplementary Table S7). These visualizations enable a straightforward comparison of phenotypic traits, enhancing the user experience and facilitating research on trait variation and selection strategies.

Our platform extends phenotypic data analysis by integrating hyperspectral data visualization with machine learning models. Hyperspectral imaging captures detailed features of plants and generates hyperspectral raw files, which undergo a structured process of data input, preprocessing, feature extraction, and classification (Supplementary Fig. S3a). Spectral extraction is carried out using MATLAB to generate corresponding .xlsx files, which are processed through predictive models for pecan variety classification. Users can upload hyperspectral data via a dedicated submission form (Supplementary Table S6) without creating an account, and the backend employs machine learning models to analyze the spectral data. Upon successful submission, the model processes the data immediately, eliminating the need for long wait times or complex coding (Supplementary Fig. S3b). The predicted variety classifications are displayed directly on the web page (Supplementary Fig. S3c), providing a seamless and efficient workflow for users. Hyperspectral imaging and machine learning integration provide advanced research tools, enhancing data interpretation and supporting breeding and efficient cultivar development.

### Case study: exploring WRINKLED1 functions across species using DEFGermplasm tools

DEFGermplasm aims to establish a comprehensive, one-stop forest germplasm genomic resource and analysis platform. Here, we conduct an in-depth bioinformatics analysis using the WRINKLED1 (WRI1) transcription factor as an example to demonstrate the platform's capabilities.

We may have an interesting WRI1 gene, such as AT3G54320.3, that we want to study. To do so, we can start by identifying orthologous genes using the cross-species alignment tool (Supplementary Fig. S4). This tool allows us to search *Arabidopsis*, Chinese fir, hickory, pecan, phoebe, and poplar databases. The search reveals 62 potential orthologous genes related to WRI1, each with detailed information that can be downloaded using the 'Download' button in the search results table (Supplementary Fig. S4a, Supplementary Table S8).

With the aid of Pfam annotations, we can see the pecan's orthologous genes (Ci.v01.chr13G04270 and Ci.v01.chr16G07600) related to WRI1, as shown in the search results table. Further exploration of the table reveals additional orthologous genes in hickory (CCA0578S0089) and other species. The tool also provides access to detailed sequence information, enabling further investigation into gene structures or a homology search analysis to be conducted through the genome browser by clicking on the gene IDs (Supplementary Fig. S4b).

The expression atlas intuitively visualizes the expression of WRI1 across different species' tissues by navigating the tissue buttons on the left. In this case, one of the WRI1 expressions is shown to increase during pecan nut and hickory embryo development (Supplementary Fig. S4c), aligning with its well-characterized function as a key transcriptional regulator of fatty acid biosynthesis and seed oil accumulation^[100]^. In forest species, seed oil content plays a critical role in determining the economic value of species such as hickory and pecan, making WRI1 an essential target for breeding programs aimed at improving lipid accumulation in nuts.

Further examination reveals strong WRI1 expression in pecan flowers and mild expression in the hickory stigma and Chinese fir cones, suggesting a potential role in the reproductive organ development of angiosperm and gymnosperm trees. WRI1 is also expressed in Chinese fir, pecan, and phoebe leaves, suggesting a role in cuticular wax biosynthesis (Supplementary Fig. S4d). Cuticular wax is crucial for desiccation tolerance and stress adaptation in gymnosperms and angiosperms^[101]^. The findings indicate that WRI1 exhibits functional conservation across diverse taxa, whereas in gymnosperms WRI1 mostly involves leaf wax formation.

Detailed expression data for each tissue are displayed in a table format for comprehensive analysis. Based on cross-species comparisons, we can return to species-specific expression profiles (Supplementary Fig. S4e) to gain a broader perspective on gene information. This integrated approach facilitates further analysis using the platform's tools, supporting breeding and conservation efforts.

## Discussion

This study introduces DEFGermplasm, a digital platform designed to address challenges in integrating, visualizing, and analyzing forest tree germplasm resources. The platform currently includes pecan, hickory, phoebe, and Chinese fir, and there are plans to expand it with additional species in the future. By leveraging high-throughput sequencing and bioinformatics advancements, DEFGermplasm provides researchers with comprehensive genomic and phenotypic data analysis tools to support forest genetics, breeding, and conservation efforts.

### Comparison with existing genomics platforms

Several genomic databases serve plant and forest research communities, each with distinct strengths and focuses. For instance, Phytozome (https://phytozome-next.jgi.doe.gov) provides high-quality genome assemblies and annotations for a wide range of plant species but lacks extensive phenotypic data and forest-specific tools. TreeGenes (treegenesdb.org) specializes in the genetic diversity and population genomics of forest trees, offering genetic markers and GWAS tools, although its phenotypic data are limited to a few traits (e.g., drought tolerance). PlantGenIE (http://plantgenie.org/) focuses on gene regulatory networks and expression atlases, although it covers few tree species and lacks comprehensive phenotypic datasets. DEFGermplasm bridges these gaps by integrating genomic, transcriptomic, and phenotypic datasets alongside hyperspectral imaging. While existing tree genomics platforms primarily emphasize data retrieval, DEFGermplasm provides a comprehensive analysis workflow, including gene discovery, expression analysis, and cross-species comparisons, using a comparative genomics tool. Additionally, its intuitive visualization interface enhances usability for breeding and conservation applications. DEFGermplasm will continue to expand its species coverage, enrich its functional modules, and incorporate advanced analytical tools such as deep learning to meet growing research needs. As data volumes and user demands increase, we plan to optimize the platform further to enhance system responsiveness, efficiency, and real-time interaction capabilities in large-scale data environments, ensuring that DEFGermplasm remains a valuable resource for forest genomics, breeding, and conservation applications.

### Cross-species expression and functional analysis

One of the key features of DEFGermplasm is its Expression Atlas tool, which allows researchers to explore and compare gene expression patterns across pecan, hickory, phoebe, and Chinese fir. This capability helps in identifying tissue-specific traits and supports cross-species analyses. Interactive features such as tissue selection and advanced filtering make it easier to identify highly expressed genes, offering valuable insights for functional studies. With flexible search options and downloadable results, the tool supports efficient transcriptomic analysis and enhances the understanding of gene function.

The Cross-Species Expression Atlas further expands this capability by enabling comparisons of gene expression patterns across species, revealing conserved and divergent gene family traits. By integrating tissue heat maps, fuzzy searches, and interactive visualizations, it simplifies the exploration of orthologous gene expression and regulatory mechanisms.

This utility was demonstrated in cross-species analyses of WRI1, a key regulator of fatty acid metabolism and seed development^[102,103]^. The tool identified 62 orthologous WRI1 genes across *Arabidopsis*, Chinese fir, hickory, pecan, phoebe, and poplar, with sequence data and annotations supporting their classification. Expression heat maps showed high expression of WRI1 during pecan and hickory nut development, aligning with its role in fatty acid biosynthesis^[104]^. Researchers can use this information to identify highly expressed WRI1 genes in pecan and hickory, which are potentially linked to higher seed oil content, an economically important trait. These findings assist in marker-assisted selection and natural population screening, helping conservationists select diverse and valuable germplasms for seed banks.

Beyond seed development, high WRI1 expression in Chinese fir, pecan, and phoebe leaves suggests a role in cuticular wax formation an important factor for desiccation resistance and pathogen defense^[105]^ in both gymnosperms and angiosperms. Additionally, WRI1 expression in reproductive tissues, including pecan flowers, hickory stigma, and Chinese fir cones, highlights its potential regulatory roles in reproductive development. This information enables researchers to explore WRI1-expressing genotypes linked to environmental adaptation, with implications for afforestation projects and conservation strategies.

Overall, the results highlight the functional conservation of WRI1 across diverse plant taxa, suggesting that its role extends beyond oil biosynthesis^[100]^ and may contribute to reproductive success and adaptation in different ecological niches. These findings provide a foundation for studying gene conservation, functional adaptation, and their potential applications in breeding programs and conservation efforts.

### Genomic tools for functional studies

Beyond expression analysis, DEFGermplasm supports functional studies through its sequence alignment and BLAST tools. These tools efficiently identify sequence homology and orthologous genes across species, integrating datasets from pecan, hickory, Chinese fir, phoebe, and other species. Customizable parameters and detailed graphical outputs, such as chord diagrams and alignment summaries, enable researchers to visualize sequence relationships and explore gene functions. Together with downloadable data and genome visualization, this tool supports research on gene conservation, evolution, and functional adaptation.

To complement these tools, the platform integrates with JBrowse, providing an intuitive interface for genome structure and annotation. Researchers can examine gene sequences, exons, introns, and regulatory elements in detail. The functional panel delivers comprehensive information, while support for custom genome uploads enhances flexibility. Integration with other analytical tools allows seamless expression transitions and further genomic studies.

### Phenotypic and hyperspectral data visualization

The phenotypic data visualization module enhances genomic analyses by enabling comparisons of traits such as fruit morphology, physiological characteristics, and timber properties. For nut-bearing species such as pecan and hickory, it supports breeding efforts to improve yield, while for timber species such as Chinese fir and phoebe, it aids research on growth and adaptability for sustainable forestry. The integration of machine learning with hyperspectral imaging aids in identifying superior fruit varieties in field research while improving the visualization and communication of model results. Future developments will focus on expanding machine learning applications to enhance the user experience and platform functionality.

## Conclusions

DEFGermplasm is a digital platform designed to integrate, visualize, and analyze germplasm data and to support the digitization and conservation of forest resources. By providing user-friendly tools for genomic and phenotypic analysis, the platform facilitates research on genetic diversity, trait improvement, and sustainable resource management. Its scalable framework allows for the inclusion of diverse species, making it a versatile resource for advancing germplasm research and conservation efforts.

## SUPPLEMENTARY DATA

Supplementary data to this article can be found online.

## Data Availability

The genomic data for *Arabidopsis thaliana* (Araport11) and *Populus trichocarpa* (version 4.1) are available through the Phytozome repository. In addition, the datasets pertaining to pecan, hickory, Chinese fir, and phoebe that were analyzed in this study are included within this article and its supplementary information files.

## References

[1] (2014). Utilization and transfer of forest genetic resources: a global review. Forest Ecology and Management.

[2] (2010). Phytodiversity (Angiosperms and Gymnosperms) in Chaurangikhal Forest of Garhwal Himalaya, Uttarakhand, India. Indian Journal of Science and Technology.

[3] (2017). Biodiversity and ecosystem services in forest ecosystems: a research agenda for applied forest ecology. Journal of Applied Ecology.

[4] (2000). Forest functions, ecosystem stability and management. Forest Ecology and Management.

[5] (2020). Functional and morphological evolution in gymnosperms: a portrait of implicated gene families. Evolutionary Applications.

[6] (2007). Bioenergy potentials from forestry in 2050. Climatic Change.

[7] (2023). Exploring the seasonal dynamics and molecular mechanism of wood formation in gymnosperm trees. International Journal of Molecular Sciences.

[8] (2012). Ecological and genetic factors linked to contrasting genome dynamics in seed plants. New Phytologist.

[9] (2009). Darwin's second 'abominable mystery': Why are there so many angiosperm species. American Journal of Botany.

[10] (2004). The origin and diversification of angiosperms. American Journal of Botany.

[11] (2018). Agroforestry systems: Meta-analysis of soil carbon stocks, sequestration processes, and future potentials. Land Degradation & Development.

[12] (2017). The sexual advantage of looking, smelling, and tasting good: the metabolic network that produces signals for pollinators. Trends in Plant Science.

[13] (2019). Metabolomic and gene expression approaches reveal the developmental and environmental regulation of the secondary metabolism of yacón (*Smallanthus sonchifolius*, Asteraceae). Scientific Reports.

[14] (2019). Improving production of plant secondary metabolites through biotic and abiotic elicitation. Journal of Applied Research on Medicinal and Aromatic Plants.

[15] (2002). Plant cell cultures: chemical factories of secondary metabolites. Biotechnology Advances.

[16] (2018). The sequenced angiosperm genomes and genome databases. Frontiers in Plant Science.

[17] (2018). Climate change impacts on boreal forest timber supply. Forest Policy and Economics.

[18] (1998). Global climate change and tropical forest genetic resources. Climatic Change.

[19] (2019). Editorial: forest health under climate change: effects on tree resilience, and pest and pathogen dynamics. Frontiers in Plant Science.

[20] (2015). Increasing human dominance of tropical forests. Science.

[21] (2017). Big data for forecasting the impacts of global change on plant communities. Global Ecology and Biogeography.

[22] (2021). Germplasm conservation: instrumental in agricultural biodiversity—a review. Sustainability.

[23] (2012). Challenges and opportunities with big data. Proceedings of the VLDB Endowment.

[24] (2021). The digital forest: mapping a decade of knowledge on technological applications for forest ecosystems. Earth's Future.

[25] (2022). Potential for artificial intelligence (AI) and machine learning (ML) applications in biodiversity conservation, managing forests, and related services in India. Sustainability.

[b26] 26Vertakova Y, Agamagomedova S, Sergeeva I, Tarasov AV, Morkovina S, et al. 2022. Digital mechanisms of management system optimization in the forest industry. In Research Anthology on Ecosystem Conservation and Preserving Biodiversity. Hershey, PA: IGI Global Scientific Publishing. pp. 424−42. doi: 10.4018/978-1-6684-5678-1.ch022

[27] (1997). Linking modelling and visualisation for natural resources management. Environment and Planning B: Planning and Design.

[28] (2018). Redefining the role of admixture and genomics in species conservation. Conservation Letters.

[29] (2018). Genetic diversity and conservation units: dealing with the species-population continuum in the age of genomics. Frontiers in Ecology and Evolution.

[30] (2010). Genomics and the future of conservation genetics. Nature Reviews Genetics.

[31] (2022). PhenoApp: a mobile tool for plant phenotyping to record field and greenhouse observations. F1000Research.

[32] (2022). Phenotypic traits extraction and genetic characteristics assessment of *Eucalyptus* trials based on UAV-borne LiDAR and RGB images. Remote Sensing.

[33] (2022). Digital extended specimens: enabling an extensible network of biodiversity data records as integrated digital objects on the Internet. Bioscience.

[34] (2019). Comparing different supervised machine learning algorithms for disease prediction. BMC Medical Informatics and Decision Making.

[35] (2021). Machine learning for large-scale crop yield forecasting. Agricultural Systems.

[36] (2020). Blockchain data-based cloud data integrity protection mechanism. Future Generation Computer Systems.

[b37] 37Lai K. 2016. WheatGenome.info: a resource for wheat genomics resource. In Plant Bioinformatics, ed. Edwards D. Vol. 1374. New York, NY:Humana Press. pp. 203−13. doi: 10.1007/978-1-4939-3167-5_10

[38] (2008). The Rice Annotation Project Database (RAP-DB): 2008 Update. Nucleic Acids Research.

[39] (2007). CSRDB: a small RNA integrated database and browser resource for cereals. Nucleic Acids Research.

[b40] 40Harper L, Gardiner J, Andorf C, Lawrence CJ. 2016. MaizeGDB: the maize genetics and genomics database. In Plant Bioinformatics, ed. Edwards D. Vol. 1374. New York, NY: Humana Press. pp. 187−202. doi: 10.1007/978-1-4939-3167-5_9

[41] (2010). SoyBase, the USDA-ARS soybean genetics and genomics database. Nucleic Acids Research.

[42] (2018). Sustaining the future of plant breeding: the critical role of the USDA-ARS national plant germplasm system. Crop Science.

[43] (2018). Design and implementation of the information sharing platform of forest germplasm resources in the Bailong River and the Taohe River forest areas. Journal of Sichuan Forestry Science Technology.

[44] (2018). Pecan nuts: a review of reported bioactivities and health effects. Trends in Food Science & Technology.

[45] (1999). Juglandaceae. Flora of China.

[46] (1979). A study of the genus Carya Nutt. in China. Acta Phytotaxonomica Sinica.

[47] (2019). Chinese fir (*Cunninghamia Lanceolata*) a green gold of china with continues decline in its productivity over the successive rotations: a review. Applied Ecology Environmental Research Letters.

[48] (2013). The regulation of cambial activity in Chinese fir (*Cunninghamia lanceolata*) involves extensive transcriptome remodeling. New Phytologist.

[49] (2016). Thinning increases understory diversity and biomass, and improves soil properties without decreasing growth of Chinese fir in Southern China. Environmental Science and Pollution Research.

[50] (2015). Development of EST-SSR markers and analysis of genetic diversity in natural populations of endemic and endangered plant *Phoebe chekiangensis*. Biochemical Systematics and Ecology.

[51] (2017). Complete chloroplast genome sequences of two endangered *Phoebe* (Lauraceae) species. Botanical Studies.

[52] (2013). Inhibition effect of extraction from seed of *Phoebe bournei*. Advanced Materials Research.

[53] (2015). Efficient way of web development using python and flask. International Journal of Advanced Research in Computer Science.

[54] (2018). Quantitative genetics and genomics converge to accelerate forest tree breeding. Frontiers in Plant Science.

[55] (2016). Application of genomic technologies to the breeding of trees. Frontiers in Genetics.

[56] (2023). Identification of *WUSCHEL*-related homeobox (*WOX*) gene family members and determination of their expression profiles during somatic embryogenesis in *Phoebe bournei*. Forestry Research.

[57] (2019). The genomes of pecan and Chinese hickory provide insights into *Carya* evolution and nut nutrition. GigaScience.

[58] (2022). The chromosome-scale genome of *Phoebe bournei* reveals contrasting fates of terpene synthase (TPS)-a and TPS-b subfamilies. Plant Communications.

[59] (2023). Transcriptional regulation of female and male flower bud initiation and development in pecan (*Carya* illinoensis). Plants.

[60] (2022). Integrated transcriptome and proteome analysis of developing embryo reveals the mechanisms underlying the high levels of oil accumulation in *Carya cathayensis* Sarg. Tree Physiology.

[61] (2022). Global transcriptome analysis revealed the molecular regulation mechanism of pigment and reactive oxygen species metabolism during the stigma development of *Carya cathayensis*. Frontiers in Plant Science.

[62] (2021). Full-length transcriptomic identification of R2R3-MYB family genes related to secondary cell wall development in *Cunninghamia lanceolata* (Chinese fir). BMC Plant Biology.

[63] (2014). Molecular and physiological responses to abiotic stress in forest trees and their relevance to tree improvement. Tree Physiology.

[64] (2019). Plant phenotyping: past, present, and future. Plant Phenomics.

[65] (2024). Quality detection and variety classification of pecan seeds using hyperspectral imaging technology combined with machine learning. Journal of Food Composition and Analysis.

[66] (2004). Accurate determination of moisture content of organic soils using the oven drying method. Drying Technology.

[67] (2008). Green procedure with a green solvent for fats and oils' determination Microwave-integrated Soxhlet using limonene followed by microwave Clevenger distillation. Journal of Chromatography A.

[68] (2015). Effect of acid hydrolysis on starch structure and functionality: a review. Critical Reviews in Food Science and Nutrition.

[69] (2010). Compositional analysis of lignocellulosic feedstocks. 1. Review and description of methods. Journal of Agricultural and Food Chemistry.

[70] (2008). Extraction of carotenoids and chlorophyll from microalgae with supercritical carbon dioxide and ethanol as cosolvent. Journal of Separation Science.

[71] (2022). GROP: a genomic information repository for oilplants. Frontiers in Plant Science.

[72] (2020). Portal of Juglandaceae: a comprehensive platform for Juglandaceae study. Horticulture Research.

[73] (2016). SeqKit: a cross-platform and ultrafast toolkit for FASTA/Q file manipulation. PLoS One.

[74] (2012). Bio.Phylo: a unified toolkit for processing, analyzing and visualizing phylogenetic trees in Biopython. BMC Bioinformatics.

[75] (2010). ANNOVAR: functional annotation of genetic variants from high-throughput sequencing data. Nucleic Acids Research.

[76] (2018). Uncovering full-length transcript isoforms of sugarcane cultivar Khon Kaen 3 using single-molecule long-read sequencing. PeerJ.

[77] (2000). EMBOSS: the European molecular biology open software suite. Trends in Genetics.

[78] (2018). The 'TranSeq' 3'-end sequencing method for high-throughput transcriptomics and gene space refinement in plant genomes. The Plant Journal.

[79] (2018). fastp: an ultra-fast all-in-one FASTQ preprocessor. Bioinformatics.

[80] (2014). Trimmomatic: a flexible trimmer for Illumina sequence data. Bioinformatics.

[81] (2013). STAR: ultrafast universal RNA-seq aligner. Bioinformatics.

[82] (2011). RSEM: accurate transcript quantification from RNA-Seq data with or without a reference genome. BMC Bioinformatics.

[83] (2006). Review: a gentle introduction to imputation of missing values. Journal of Clinical Epidemiology.

[84] (2015). On enhanced interquartile range charting for process dispersion. Quality and Reliability Engineering International.

[85] (2012). Data Processing with Pandas. Login.

[86] (2003). Analysis of microarray data using Z score transformation. The Journal of Molecular Diagnostics.

[87] (2010). Improving your data transformations: applying the box-cox transformation. Practical Assessment, Research & Evaluation.

[88] (2015). Hyperspectral imaging in medicine: image pre-processing problems and solutions in Matlab. Journal of Biophotonics.

[89] (2014). Estimating soil organic carbon using VIS/NIR spectroscopy with SVMR and SPA methods. Remote Sensing.

[90] (2007). Hyperspectral image compression using JPEG2000 and principal component analysis. IEEE Geoscience and Remote Sensing Letters.

[91] (2023). JBrowse 2: a modular genome browser with views of synteny and structural variation. Genome Biology.

[92] (2019). Sequenceserver: a modern graphical user interface for custom BLAST databases. Molecular Biology and Evolution.

[93] (2021). Twelve years of SAMtools and BCFtools. GigaScience.

[94] (2019). OrthoFinder: phylogenetic orthology inference for comparative genomics. Genome Biology.

[95] (2019). eggNOG 5.0: a hierarchical, functionally and phylogenetically annotated orthology resource based on 5090 organisms and 2502 viruses. Nucleic Acids Research.

[96] (2021). Seaborn: statistical data visualization. Journal of Open Source Software.

[97] (2018). ECharts: a declarative framework for rapid construction of web-based visualization. Visual Informatics.

[98] (2007). Bootstrap methods and applications. IEEE Signal Processing Magazine.

[b99] 99Christudas B. 2019. Install, configure, and run nginx reverse proxy. In Practical Microservices Architectural Patterns Berkeley, CA: Apress. pp. 843–46. doi: 10.1007/978-1-4842-4501-9_22

[100] (2019). WRINKLED1, a "master regulator" in transcriptional control of plant oil biosynthesis. Plants.

[101] (2017). Molecular and evolutionary mechanisms of cuticular wax for plant drought tolerance. Frontiers in Plant Science.

[102] (2010). Physiological and developmental regulation of seed oil production. Progress in Lipid Research.

[103] (2022). BLISTER promotes seed maturation and fatty acid biosynthesis by interacting with WRINKLED1 to regulate chromatin dynamics in *Arabidopsis*. The Plant Cell.

[104] (2016). The mechanism of high contents of oil and oleic acid revealed by transcriptomic and lipidomic analysis during embryogenesis in *Carya cathayensis* Sarg. BMC Genomics.

[105] (2003). Biosynthesis and secretion of plant cuticular wax. Progress in Lipid Research.

