# Supplementary material for: DEFGermplasm: a comprehensive digital platform for forest genomic and phenotype data integration
Source: For Res (Fayettev). 2025 May 16;5:e009. doi: 10.48130/forres-0025-0009 (PMC12434696; doi:10.48130/forres-0025-0009)
Supplement: Supplementary file 1 — Supplementary data to this article can be found online. [file forres-0025-0009-Supplementary.zip › 10.48130_forres-0025-0009-Suppl-FigureS1.pdf]

a

SequenceServer 2.0.0 using **BLASTP 2.15.0+**, query submitted on 2025-04-22 07:48:24 UTC
Databases: Annamocarya_sinensis, Arabidopsis_Araport11, Caray_illinoensis, Carya_cathayensis_Sarg, CillinoinensisPawnee 588 v 1.0, Cyclocarya_paliurus, Juglans_cathayensis, Juglans_cinerea, Juglans_hindsii, Juglans_hopeiensis, Juglans_major, Juglans_mandshurica, Juglans_nigra, Juglans_regia, Juglans_sigillata, Phoebe_bournei, Platycarya_strobilacea, Populus_v4.1, chinese fir (325972 sequences, 119920468 characters)
Parameters: evalue 1e-05, matrix BLOSUM62, gap-open 11, gap-extend 1, filter F
Please cite: https://doi.org/10.1093/molbev/msz185

b

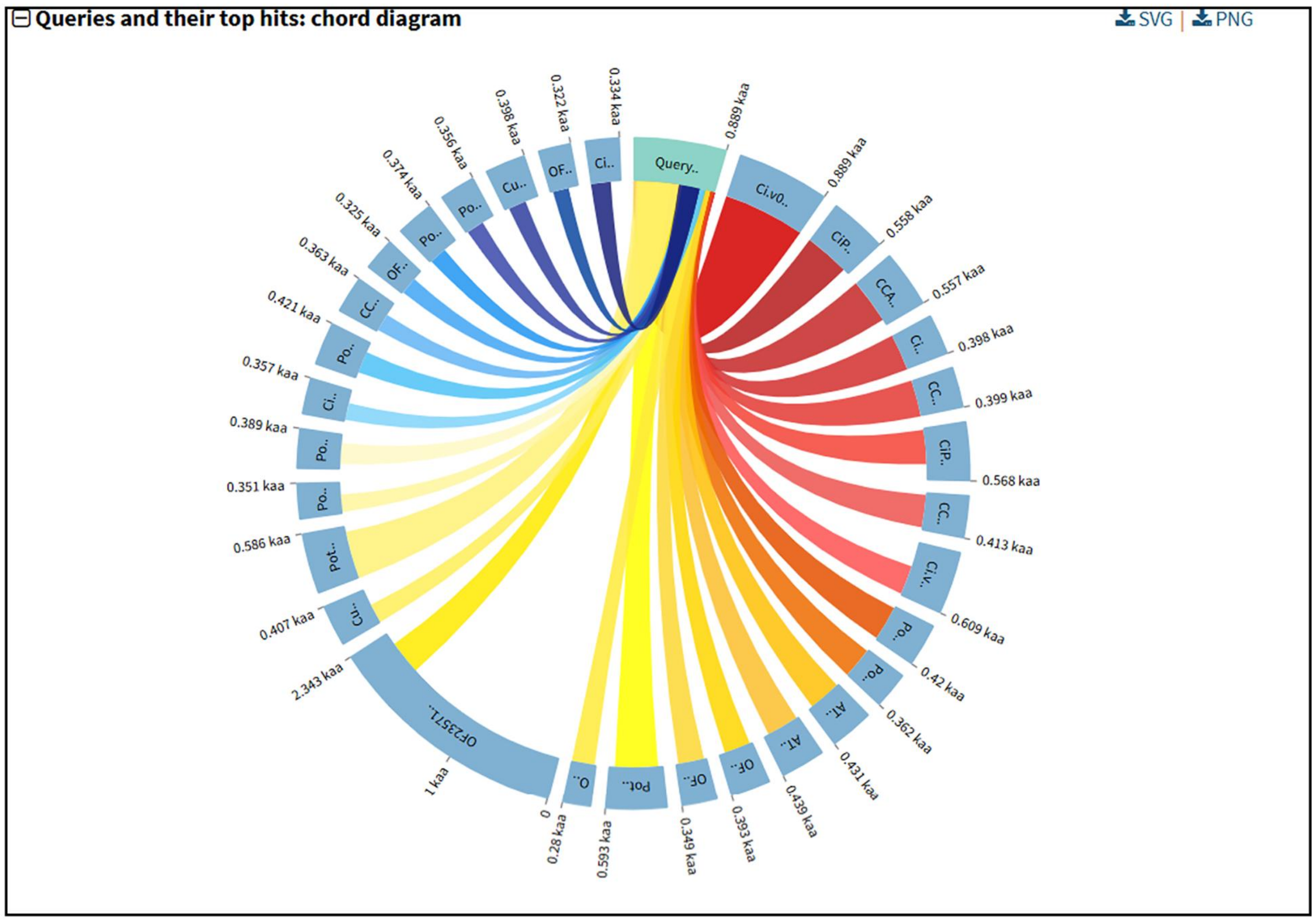

c

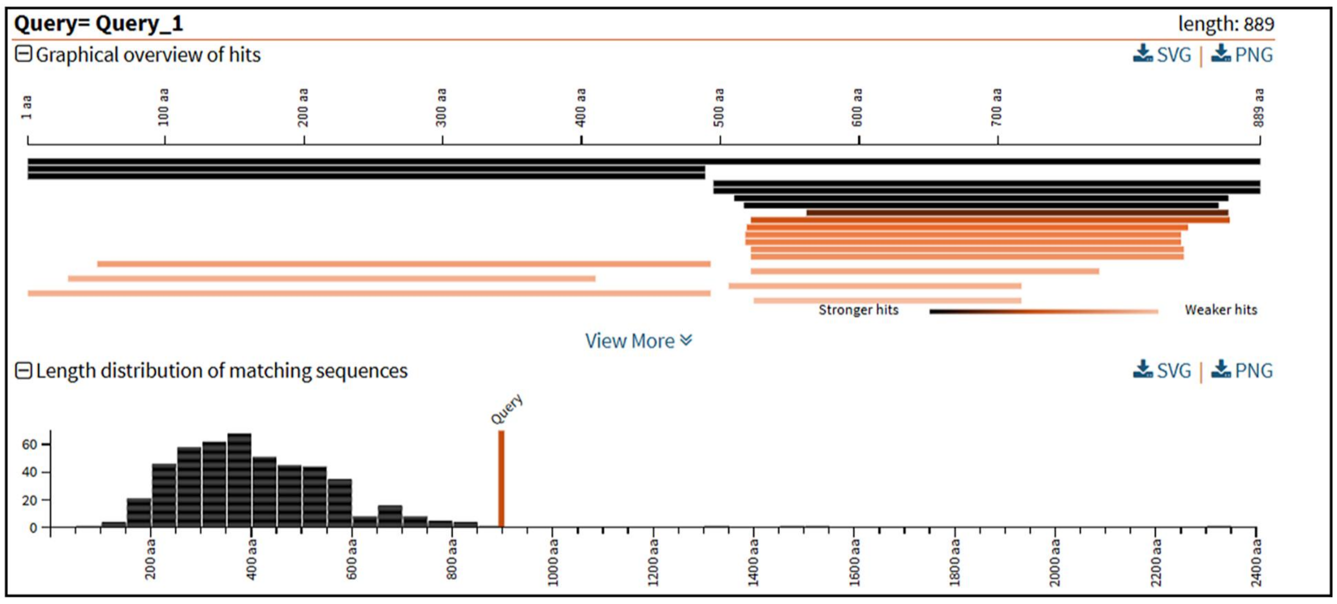

d

Sequences producing significant alignments

#	Similar sequences	Query coverage (%)	Total score	E value	Identity (%)
1.	Ci.v01.chr16G07600	100	4783	0	100%
2.	CiPaw.13G047300	55	2569	0	100%
3.	CCA1573S0031	55	2493	0	97.5%
4.	CiPaw.13G047400	44	2160	0	99.2%
5.	CCA1573S0032	44	2102	0	97.5%
6.	CiPaw.14G037700	40	1374	4.55×10^{-180}	75.5%
7.	CCA0578S0089	39	1350	8.11×10^{-179}	77.2%
8.	Ci.v01.chr13G04270	34	1223	7.74×10^{-157}	75.9%
9.	Potri.010G247200.1	39	1038	4.88×10^{-132}	62%
10.	Potri.008G011900.1	36	960	3.22×10^{-121}	64%
11.	AT3G54320.3	35	918	4.89×10^{-114}	55.7%
12.	AT3G54320.1	35	917	7.73×10^{-114}	55.6%
13.	OF28243	35	887	5.39×10^{-110}	60.2%
14.	OF11739	35	867	1.08×10^{-107}	59.3%
15.	Potri.010G247100.1	50	860	1.99×10^{-103}	49.9%
16.	OF23570	28	810	2.53×10^{-100}	66.7%
17.	OF23571	43	867	2.31×10^{-97}	53.3%
18.	Cunninghamia90205	24	801	2.71×10^{-97}	71.2%
19.	Potri.008G012000.1	55	816	3.22×10^{-97}	47.4%
20.	Potri.003G185300.1	22	764	1.05×10^{-92}	73.1%

Supplementary_Figure_S1: Overview of sequence alignment results and visualization features provided by the DEFGermplasm platform. (a) Summary of the alignment settings used in the analysis, including the BLAST version, selected reference database, and user-defined parameters such as mismatch tolerance. (b) Chord diagram displaying the relationships between query sequences and their top matching sequences across different species, illustrating the degree of sequence similarity. (c) Visualization of alignment hits and the length distribution of matched sequences, offering intuitive insights into alignment quality and coverage. (d) Tabular presentation of detailed alignment statistics for each query–target pair, including query coverage, total score, E-value, and identity percentage, enabling precise evaluation of homology strength.
