# Supplementary material for: DEFGermplasm: a comprehensive digital platform for forest genomic and phenotype data integration
Source: For Res (Fayettev). 2025 May 16;5:e009. doi: 10.48130/forres-0025-0009 (PMC12434696; doi:10.48130/forres-0025-0009)
Supplement: Supplementary file 1 — Supplementary data to this article can be found online. [file forres-0025-0009-Supplementary.zip › 10.48130_forres-0025-0009-Suppl-FigureS2.pdf]

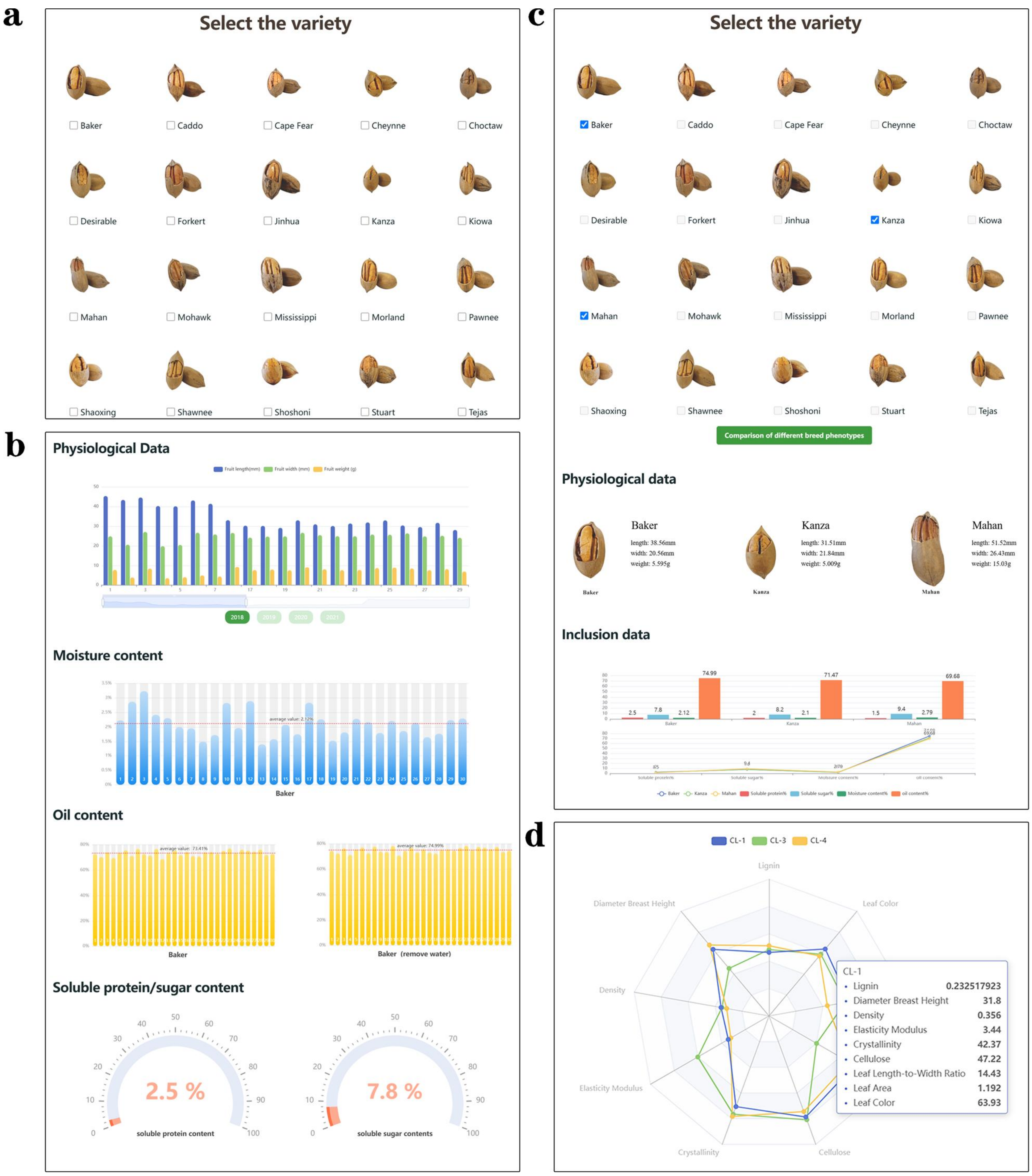

Supplementary_Figure_S2: Visualization of phenotypic traits. (a) Comprehensive phenotypic visualization of pecan varieties. (b) Interactive bar charts showing detailed metrics, including fruit moisture content, oil content, soluble protein, and soluble sugar. (c) Comparative analysis of phenotypic traits and internal substance contents (moisture, oil, soluble protein, and soluble sugar) for three selected pecan varieties. (d) Radar chart visualization of the phenotype data for Chinese fir, including traits such as lignin content, diameter at breast height, density, elasticity modulus, crystallinity, cellulose content, leaf length-to-width ratio, leaf area, and color value.
