# Supplementary material for: DEFGermplasm: a comprehensive digital platform for forest genomic and phenotype data integration
Source: For Res (Fayettev). 2025 May 16;5:e009. doi: 10.48130/forres-0025-0009 (PMC12434696; doi:10.48130/forres-0025-0009)
Supplement: Supplementary file 1 — Supplementary data to this article can be found online. [file forres-0025-0009-Supplementary.zip › 10.48130_forres-0025-0009-Suppl-FigureS3.pdf]

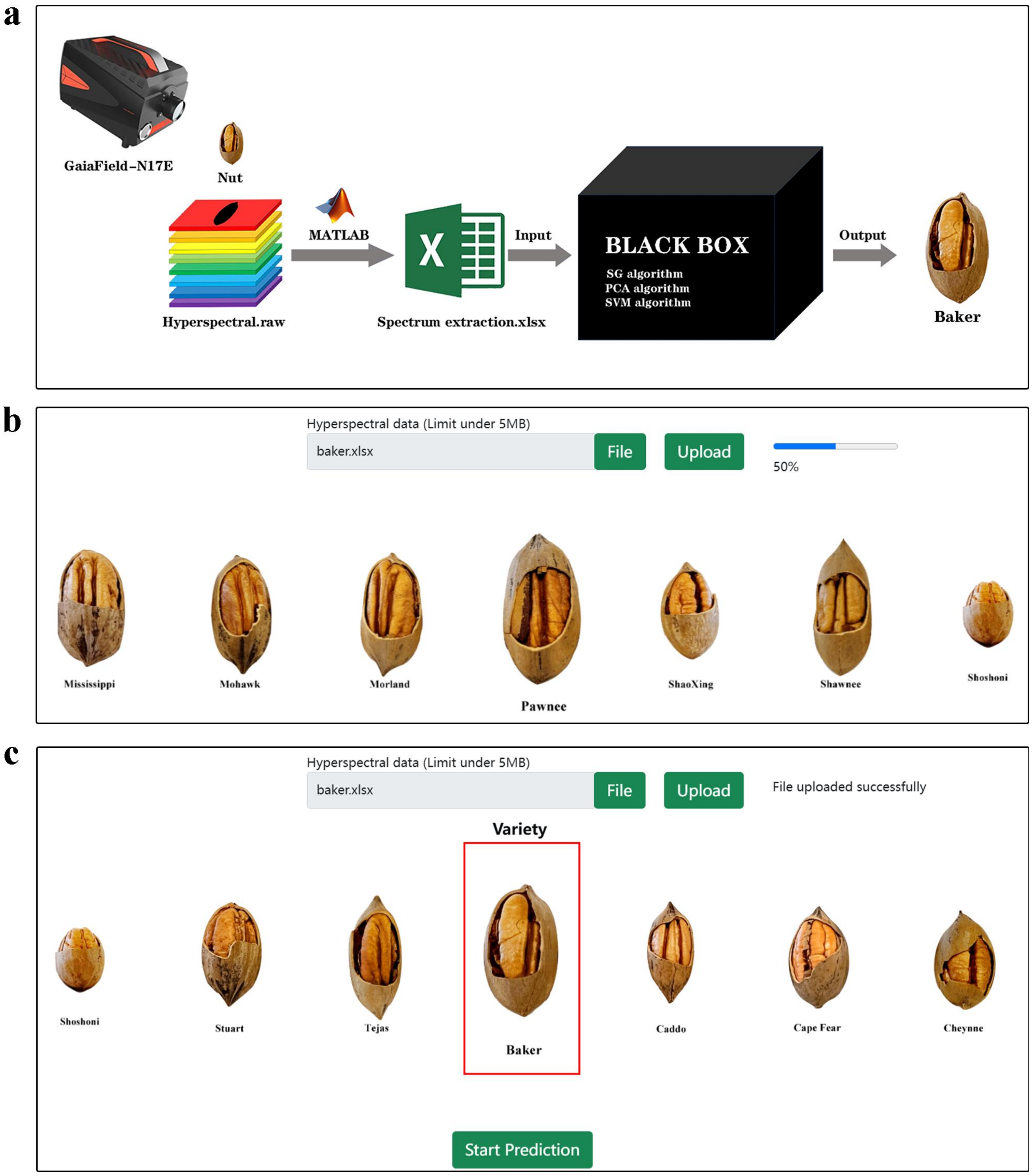

Supplementary_Figure_S3: Integration of hyperspectral imaging and machine learning for phenotypic analysis of pecan seeds. (a) The complete workflow, including hyperspectral image acquisition, preprocessing, feature extraction, model training, and final prediction output. This pipeline enables accurate, non-destructive phenotypic assessment. (b) User submission interface for uploading hyperspectral data. Users can input their datasets to perform pecan variety classification based on trained machine learning models. (c) Visualization of prediction results, presenting the identified pecan variety based on spectral data analysis.
