# Supplementary figures and images for: DEFGermplasm: a comprehensive digital platform for forest genomic and phenotype data integration
Source: For Res (Fayettev). 2025 May 16;5:e009. doi: 10.48130/forres-0025-0009 (PMC12434696; doi:10.48130/forres-0025-0009)

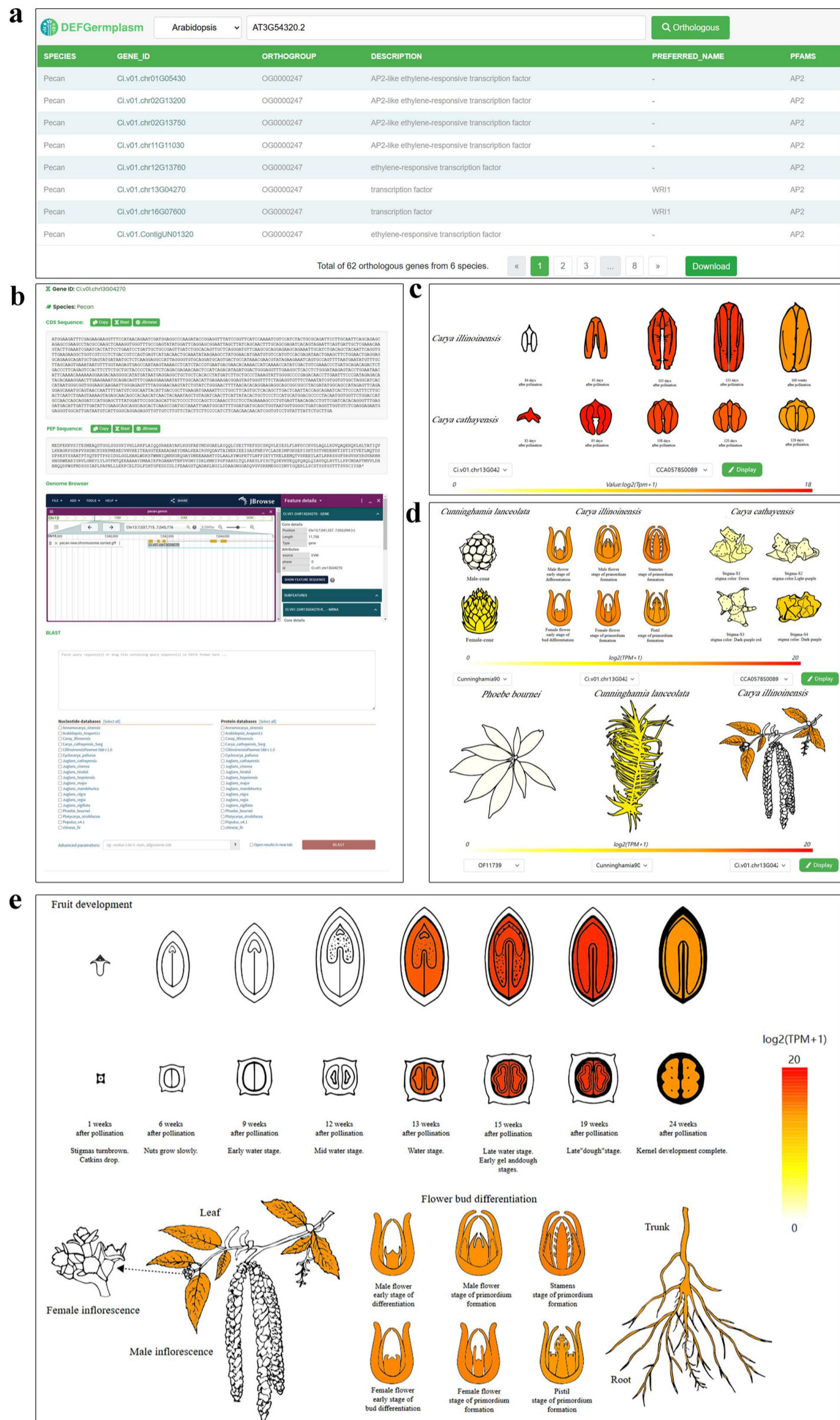

Supplement: Supplementary file 1 — Supplementary data to this article can be found online. [file forres-0025-0009-Supplementary.zip › 10.48130_forres-0025-0009-Suppl-FigureS4.pdf]
